# Immune-Mediated Vascular Injury and Dysfunction in Transplant Arteriosclerosis

**DOI:** 10.3389/fimmu.2014.00684

**Published:** 2015-01-12

**Authors:** Anna von Rossum, Ismail Laher, Jonathan C. Choy

**Affiliations:** ^1^Department of Molecular Biology and Biochemistry, Simon Fraser University, Burnaby, BC, Canada; ^2^Department of Anaesthesiology, Pharmacology and Therapeutics, University of British Columbia, Vancouver, BC, Canada

**Keywords:** organ transplantation, transplant arteriosclerosis, blood vessels, endothelium, T cell, antibodies

## Abstract

Solid organ transplantation is the only treatment for end-stage organ failure but this life-saving procedure is limited by immune-mediated rejection of most grafts. Blood vessels within transplanted organs are targeted by the immune system and the resultant vascular damage is a main contributor to acute and chronic graft failure. The vasculature is a unique tissue with specific immunological properties. This review discusses the interactions of the immune system with blood vessels in transplanted organs and how these interactions lead to the development of transplant arteriosclerosis, a leading cause of heart transplant failure.

## Introduction

The success of organ transplantation as a curative therapy is hindered by the eventual failure of almost all grafts due largely to immune-mediated rejection (1). Also, transplant recipients need to take non-specific immunosuppressive drugs that are associated with many morbid side effects. A better understanding of how immune responses that are directed toward foreign organ grafts cause transplant failure is needed to develop strategies that specifically prolong survival and increase quality of life of graft recipients.

By its nature, organ transplantation results in the exposure of the immune system to an abundance of foreign antigens associated with inflammation, the former being a result of genetic differences between organ donors and recipients and the latter being caused by ischemic and mechanical damage during the transplantation procedure. The result is that T and B cells of the adaptive immune system specifically recognize graft-derived antigens (an alloimmune response) and become activated to elicit effector responses that reject transplanted organs. T cells recognize graft-derived peptides bound to major histocompatibility complexes [pMHCs; human leukocyte antigens (HLAs) in humans] expressed by graft cells and recipient antigen-presenting cells (e.g., dendritic cells and macrophages). This leads to the induction of cell- and antibody-mediated alloimmune responses (2, 3). Alloimmune-mediated arterial injury and dysfunction causes the development of transplant arteriosclerosis (TA), a vascular occlusive condition that causes ischemic graft failure. TA is prevalent in all solid organ transplants and is the main challenge in heart transplants because its incidence has not been diminished by advancements in current immunosuppressive drug therapies despite their ability to prevent acute rejection (1, 4). This may be because the immune response in arteries has unique features that necessitate distinct approaches for intervention. We review the current knowledge on the mechanisms by which alloimmune responses lead to vascular cell injury and dysfunction, the alarmin molecules released in response to alloimmune-mediated cell injury, and how these processes drive the development of TA.

## Immune Targeting of Vascular Cells

In response to T cell recognition of pMHC molecules, T cells undergo rapid activation, proliferation, and differentiation into effector cells. Effector CD8 T cells are equipped to specifically induce cell death of target cells by expressing cytotoxic molecules, such as granzymes and perforin, which are contained within cytotoxic granules. Death ligands, such as FasL and TRAIL, are also expressed on CD8 T cell membranes (5–8). In addition to cytotoxic effector mechanisms, CD8 T cells also secrete interferon-γ (IFNγ), which induces cellular changes that lead to tissue remodeling. Effector properties of CD4 T cells mainly involve the production of cytokines to induce inflammation and that alter cell function in tissues. Three main types of CD4 effector T cells have been described in peripheral tissues: Th1 (that produce IFNγ), Th2 (that produce IL-4, IL-5, and IL-13), and Th17 (that produce IL-17, IL17F, IL-21, and IL-22) (9). Follicular helper T cells (Tfh; that produce IL-21 and express ICOS and CXCR5) reside within lymphoid tissues and control antibody production and class switching (10). Finally, antibodies that are secreted by B cells bind antigens within tissues and damage cells through complement-mediated cell lysis, activation of inflammation, and antibody-dependent cell cytotoxicity that is mediated by natural killer (NK) cells (11).

In addition to providing a conduit for tissue oxygenation and delivery of nutrients, blood vessels also interact intimately with the immune system to control the outcome of immune responses. Endothelial changes in the microvasculature are essential for leukocyte migration into sites of inflammation. Human endothelial cells (ECs) also basally express MHC class I and II molecules and up-regulate both molecules in response to inflammatory cytokines such as IFNγ and TNF, thereby enabling them to directly present alloantigens to T cells and be targeted by alloreactive effector T cells (12–16). Further, this vascular cell type expresses a variety of co-stimulatory molecules and, as such, human ECs are able to directly activate alloreactive memory T cells within the vessel wall (17). In addition to ECs, most arteries also contain resident dendritic cells that elicit immune activation after activation by inflammatory stimuli (18–20). Importantly, animal models have established that antigen presentation by both human ECs and arterial dendritic cells activate immunopathological T cell responses within arteries that lead to arteriosclerotic thickening (21–23). In contrast to activating T cells, the endothelium also expresses some immunoregulatory cell surface and soluble factors that inhibit effector T cell responses and some types of dendritic cells induce tolerance to arterial antigens (19, 24–29). Vascular smooth muscle cells within arteries also interact with T cells. This vascular cell type basally expresses MHC class I molecules and can be induced to express MHC class II molecules in response to inflammatory cytokines (30). T cell recognition of alloantigens presented by vascular smooth muscle cells attenuates T cell activation through the production of indoleamine 2,3-dioxygenase (IDO) and a lack of co-stimulatory molecule expression, implying that vascular smooth muscle cells possess properties that may define immunoprivilege-like status in arteries (31–33). All together, the distinct combination of immune-stimulatory and immune-regulatory features of vascular cells and artery-associated dendritic cells may define the uniqueness of immune responses in blood vessels.

Once activated by alloantigens, immune targeting of the graft vasculature occurs through processes mediated by cytotoxic CD8 T cells, effector CD4 T cells, and B cell-derived antibodies. Cytotoxic T cells induce EC death through a granzyme/perforin mechanism that is inhibited by Bcl-2. Moreover, granzyme B and perforin are sufficient to induce rapid cell death of human ECs *in vitro* and granzyme B alone is capable of inducing EC death in a delayed fashion by proteolyzing extracellular proteins required for adhesion-mediated cell survival (34–38). With regard to death receptors, vascular ECs express low levels of Fas and are relatively resistant to FasL-mediated apoptosis due to their expression of c-FLIP, which is an endogenous inhibitor of caspase-8 (39, 40). However, IFNγ and oxidized low-density lipoproteins (which are present in human TA lesions) sensitize ECs to Fas-mediated cell death by down-regulating expression of c-FLIP (41–43). The death ligand TRAIL, which is expressed by some types of T and NK cells, induces EC death *in vitro* so may also induce EC death in certain inflammatory settings (44). In addition to cytotoxic T cells, ECs also activate alloreactive CD4 T cells, which lead to the production of mainly IFNγ and IL-2, although a small subset of T cells produce IL-17 (22, 45).

B cell responses contribute to allograft injury through the production of graft-reactive antibodies (46). The presence of anti-donor antibodies is associated with a high rate of rejection and poor long-term outcome (47, 48). The histological description of antibody-mediated rejection (AMR) is vascular in nature including morphological changes to the microvascular endothelium, such as EC swelling, and the intravascular accumulation of monocytes. The observation of complement deposition in the vascular compartment of biopsies adds additional prognostic value (49). Foreign HLA molecules are the predominant antigens recognized by pathologic antibodies in the setting of transplantation but some non-HLA molecules are also targeted (50–53).

There are several cellular mechanisms by which antibodies can cause pathological changes in ECs. One of the main effector processes triggered by antibodies is complement activation. The presence of complement-binding anti-HLA antibodies is associated with extremely poor kidney graft survival as compared with the presence of non-complement-binding antibodies or the absence of donor anti-HLA antibodies (54). Also, grafts and/or recipients that are unable to activate complement fail to reject grafts in preclinical models, and therapeutic inhibition of complement with blocking antibodies prevents acute AMR in preclinical studies and clinical trials (55–59). Although vascular deposition of complement is used as a diagnostic feature of AMR, complement-mediated lysis of ECs is rarely observed (60, 61). Instead, membrane deposition of the membrane attack complex of the complement cascade augments immune responses by increasing inflammation and supporting the activation of T cells by the endothelium (62). The complement fragments C3a and C5a also have pro-inflammatory effects that increase the ability of antigen-presenting cells to activate alloreactive T cells, which oppose the induction of regulatory T cells, and that directly amplify the activation of effector T cells (63, 64).

Binding of antibodies to HLA antigens on ECs also initiates complement-independent processes that cause phenotypic changes in vascular cells. Cross-linking of HLA I molecules by antibodies triggers the downstream activation of Rho kinase and ERK1/2 signaling pathways (65). This leads to phenotypic changes that include cell proliferation, survival, and migration (66–68). HLA cross-linking also induces the rapid cell surface presentation of P-selectin and secretion of von Willebrand factor, which increases transendothelial migration of leukocytes (69, 70). Other effects of HLA cross-linking include up-regulation of cell adhesion molecules such as ICAM, chemokines such as IL-8 and RANTES, and cytokines such as IL-6 (71). This could result in prolonged activation of the endothelium that supports leukocyte recruitment and chronic inflammation.

In the discussion above, we have introduced the mechanisms by which alloimmune responses damage the graft vasculature. The effect on transplantation of these processes depends on whether the microvasculature or macrovasculature is affected. Microvascular injury results in hemorrhage and thrombosis, thereby causing ischemic graft damage that leads to acute graft failure or chronic fibrosis (72, 73). EC death also results in the release of fibrotic factors that can directly drive tissue fibrosis (74, 75). Macrovascular damage of arteries and arterioles triggers the development of TA, as discussed below (76).

## Immunopathological Mechanisms in TA

Transplant arteriosclerosis is characterized by intimal hyperplasia and vasomotor dysfunction that develops as a result of immunological targeting of vascular endothelial and smooth muscle cells. The intima in TA is formed by the accumulation of smooth muscle cells, CD4 and CD8 T cells, B cells, macrophages, dendritic cells, and occasional NK cells. Structurally, there is concentric intimal expansion, alteration of extracellular matrix composition, aberrant lipid deposition, and intraplaque hemorrhage (77–79). In addition to intimal thickening, vasodilatory function is compromised in allograft arteries (80). The combination of intimal thickening and vasodilatory dysfunction occludes the arterial lumen, resulting in reduced blood flow and ischemic damage of downstream tissues (81).

It is clear that the development of TA is driven by alloimmune targeting of the graft vasculature because intimal thickening is confined to the graft and does not develop in experimental models in which grafts are placed in genetically identical animals or in recipients that lack adaptive immune responses (82, 83). Targeting of graft arteries by T cells and antibodies causes the development of intimal thickening through the induction of vascular cell injury, and cytokine- and antibody-mediated alteration of graft vascular cell phenotypes. T cells also cause vasoregulatory dysfunction of allograft arteries. These processes are discussed below and summarized in the Table 1.

**Table 1:** **Immunological effects on vascular cells and their consequences to arterial structure and function in TA**.

Vascular cell type	Type of immune-mediated cell death or phenotypic alteration	Consequence
Endothelial Cell	Granzyme/perforin-induced death	Reparative response leading to arteriosclerotic thickening
	FasL-mediated death	Reparative response leading to arteriosclerotic thickening?
	Cell activation by inflammatory cytokines	Vascular inflammation and leukocyte accumulation leading to arteriosclerotic thickening
	Complement-dependent antibody-mediated changes	Augmentation of antigen presentation leading to arteriosclerotic thickening
	Complement-independent antibody-mediated changes	Vascular inflammation, leukocyte accumulation, and cell proliferation potentially leading to arteriosclerotic thickening
	Alteration in NO production by inflammatory cytokines	Vasoregulatory dysfunction or compensatory vasodilation
Vascular smooth muscle	FasL-mediated cell death	Medial damage potentially leading to arteriosclerotic thickening and reduced vasoconstriction
	ET-1-induced contraction	Pathological vasoconstriction
	iNOS-induced NO de-sensitization	Reduced vasodilation

### Vascular cell injury in TA

Elegant studies in the 1970s and 1980s examining the response of arteries to mechanical injury demonstrated that vascular damage can initiate a “response-to-injury” process that culminates in the development of arteriosclerotic thickening (84). In these models, intimal thickening is caused by vascular damage that triggers the migration of leukocytes and platelets to regions of injury. The resultant production of cytokines and growth factors, such as PDGF and bFGF, by infiltrating leukocytes and injured vascular cells in turn stimulates smooth muscle cells to migrate into the intima and proliferate, thereby forming the nexus of hyperplastic intimal thickening (85–91). In addition to triggering the production of growth factors from infiltrating leukocytes and neighboring vascular cells, EC death also increases smooth muscle cell accumulation through the caspase-mediated generation of a bioactive fragment of the cell matrix protein perlecan, which inhibits smooth muscle cell death (52, 92). The concept that arteriosclerotic changes are driven by a reparative response in the arterial wall was proposed to be generalizable to several forms of vascular occlusive diseases, including TA (93).

Clinical and experimental findings show that immunologic injury of endothelial and/or smooth muscle cells is a main trigger for the development of TA. Detailed histopathological analysis of clinical specimens of TA initially identified the presence of apoptotic luminal ECs in these arteries and cytotoxic T cells expressing perforin were present in the subendothelial space immediately underlying dying ECs (94–96). Granzyme B and FasL were also abundant in the intima of allograft arteries with TA and their presence was correlated with increased vascular cell death (94, 97).

**Figure 1:**
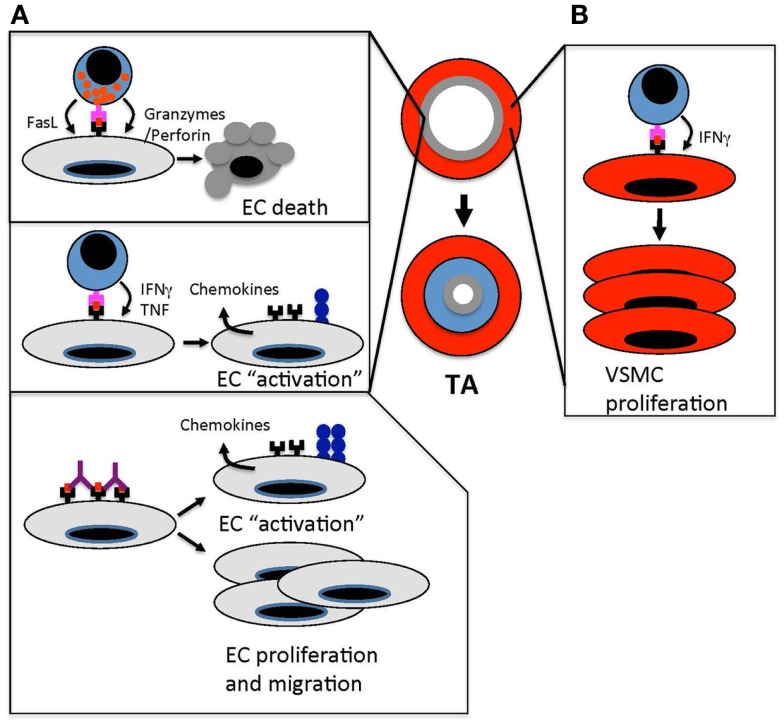
**Immune-mediated vascular changes that cause TA**. **(A)**. Effector T cells target ECs in arteries of transplanted organs. Cytotoxic T cells kill arterial ECs through granzyme/perforin- and FasL-mediated pathways. This endothelial damage triggers a “response-to-injury” process that involves leukocyte and smooth muscle cell migration into the arterial intima that drives intimal thickening and occludes the arterial lumen. In addition to cytotoxic T cell responses, T cell recognition of allogeneic ECs results in the secretion of effector cytokines, such as IFNγ and TNF, which “activates” the endothelium to up-regulate MHC class I and II, cell adhesion molecules, and chemokines. These changes amplify the recruitment of leukocytes into allograft arteries. Anti-MHC antibodies amplify immune responses in allograft arteries by cross-linking MHC molecules, which induces cell signaling pathways that up-regulate adhesion molecules and von Willebrand factor. This enhances leukocyte transendothelial migration. Anti-MHC antibodies also induce proliferation and migration of ECs, which could trigger the remodeling of allograft arteries. **(B)** The secretion of IFNγ from effector T cells stimulates the proliferation of vascular smooth muscle cells in the intima of allograft arteries. This increases the accumulation of vascular smooth muscle cells and intimal thickening.

Examination of clinical specimens of TA provides valuable insight into potential pathological mechanisms driving disease development. Pairing such observations with experimental investigations is needed to establish causative processes. Consistent with a role for EC death in the initiation of TA, early endothelial disruption characterized by missing cells, intracellular gaps, and exposed extracellular matrix is observed in arteries from heterotopic rat heart transplants very early after transplantation (98). Experiments studying the transplantation of grafts across a complete MHC barrier established a role for CD4 T cells and B cells in TA but CD8 T cells do not appear to be needed in these models (82, 83). However, it is clear in humans that many grafts reject through antibody-independent mechanisms that are mediated by T cell effector processes. The transplantation of grafts across minor histocompatibility antigen mismatch barriers leads to immunological rejection of grafts that depends on T cells but not antibodies (99). In these models, the development of TA is triggered by EC death induced by CD8 T cells, perforin, and granzyme B suggesting that this type of cytotoxic T cell response is primarily responsible for the induction of EC death in TA (Figure 1A) (37, 100–102).

Models of arteriosclerosis also suggest that medial smooth muscle cell injury can trigger or promote the development of intimal thickening (103–105). Medial smooth muscle cell death is observed in some models of arterial vascular rejection and TA, and depletion of CD8 T cells or blockade of the Fas/FasL pathway prevents medial smooth muscle cell death and intimal thickening (106, 107). Inflammatory cytokines, such as IFNγ, sensitize smooth muscle cells to FasL by relocating the Fas receptor to the cell surface (108, 109). Finally, a mechanism by which vascular smooth muscle cell death in allograft arteries triggers intimal thickening may be through the induction of stromal cell-derived factor-1 (CXCL12) production by dying and neighboring cells, which initiates the migration and proliferation of mesenchymal stem cells into the intima (110).

In addition to triggering a pathological reparative response that initiates intimal thickening, cell injury results in the release of alarmins that stimulate inflammation and immune activation. This serves to initiate or propagate immunological responses and, as such, could contribute to immunopathology. Alarmins are intracellular molecules that bind pattern recognition receptors, such as toll-like receptors (TLRs), to activate antigen-presenting cells and vascular cells (111). They also stimulate the recruitment of antigen-presenting cells to sites of injury or infection. Several alarmins have been implicated in the regulation of allogeneic responses and these include HMGB1, IL-1α, endogenous RNA and DNA, and IL-33.

HMGB1 is a chromatin binding protein that is universally expressed in cells. It is released into the extracellular space after necrotic cell death but can also be secreted in a regulated manner by macrophages. It binds to TLR4 and receptor of advanced glycation end-products (RAGE) to activate the immune stimulating properties of antigen-presenting cells (112). HMGB1 is released by dying ECs and this promotes allogeneic T cell responses by inducing the release of IL-1β from monocytes and IL-1α from neighboring ECs (113). Systemic blockade of extracellular HMGB1 prevents the development of chronic heart transplant failure and TA in a mouse model (114). IL-1α is also released from dying ECs in allograft arteries in response to ischemic and immune-mediated damage whereupon it promotes the development of intimal thickening (22). Nuclei acids are another type of endogenous molecule that is released after cellular damage and that act as alarmins. DNA and RNA from pathogens can be recognized by various TLRs as well as the STING pathway in the cytosol for DNA and the MDA5/RIG-I cytosolic RNA receptors (115). Although TLR7/8/9 may also be able to recognize self-RNA and -DNA Tellides and colleagues (116) showed that self-RNA is detected by vascular smooth muscle cells primarily through the MDA5/RIG-I pathway and that this augments inflammation within human coronary arteries.

The above examples highlight the role of alarmins in the activation of allogeneic immune responses and development of TA. Recent evidence has established a novel role for the alarmin IL-33 in preventing allogeneic immune responses, cardiac transplant failure, and TA. IL-33 is expressed in non-hematopoeitic cells including ECs, is released after cellular damage, and has been established to promote the activation of protective immune responses following virus infection (117). In contrast to the role of IL-33 in promoting immune activation in response to pathogen infection, several groups have made the unexpected observation that it prevents cardiac transplant rejection and TA (118–120). Although it promotes the development of Th2 responses, the mechanism by which IL-33 is protective in transplantation is likely through the generation of suppressive myeloid cells and T regs (120, 121).

### Cytokine-mediated alteration of graft vascular cell phenotypes

During immune responses, the endothelium is “activated” by cytokines to express cell adhesion molecules and chemokines that facilitate the recruitment of leukocytes from the blood into tissues. During this process, the endothelium also undergoes morphological changes that increase vascular permeability to plasma proteins (122). These vascular changes are essential for the development and localization of immune responses. Organ grafts are characterized by heightened inflammation and immunity resulting from ischemic and surgical damage as well as from immunological targeting of the graft. The resultant production of cytokines alters the function and phenotype of vascular cells and, in this way, remodels blood vessels (123).

Profiling of the immune response in clinical samples of TA has revealed a predominance of Th1 cytokines and associated chemokines (124). These findings suggest a pathological role of Th1 cytokines, of which IFNγ is the prototypical member, in vascular changes associated with TA. Indeed, experimental studies confirm that IFNγ has an unequivocal role in the development of TA (125, 126). IFNγ has broad-ranging effects on both ECs and vascular smooth muscle cells (Figures 1A,B). Stimulation of ECs with IFNγ up-regulates the cell surface expression of MHC class I and II molecules. This enhances the activation of T cells by graft ECs as well as the recognition and targeting of blood vessels by effector T cells (12, 127). The induction of chemokines, such as IP-10, by IFNγ supports the migration of T cells into allograft arteries (128). IFNγ also increases EC susceptibility to FasL-mediated cell death (39, 129, 130). IFNγ signaling in smooth muscle cells is also important in the development of intimal thickening, as supported by findings that IFNγ is sufficient to cause intimal thickening by promoting vascular smooth muscle cell mitogenesis in a humanized mouse model of arteriosclerosis (131). Recent studies indicate that IFNγ-stimulated smooth muscle cell proliferation is mediated by PI3K activation of mammalian target of rapamcyin (mTOR) and attenuated by ASK1-interacting protein 1, which is a Ras GTPase-activating protein family member that antagonizes JAK-STAT signaling (132, 133).

The inflammatory cytokines IL-1β, IL-1α, and TNF are also abundant in TA lesions and all are induced early after transplantation (134–136). These cytokines activate similar signaling pathways, have overlapping effects on vascular cells (137, 138), and contribute to the development of TA in animal models (22, 139). Distinctions in the effects of these cytokines may arise from differences in their cell source within arteries. While IL-1β and TNF are mainly expressed by infiltrating or tissue resident macrophages, TNF is secreted by some T cells and IL-1α is released mainly from injured parenchymal and vascular cells (140). Both TNF and IL-1 induce the rapid up-regulation of MHC molecules on ECs and support the transmigration of leukocytes into allograft arteries (137, 138). TNF can be cytotoxic to vascular cells in some situations (141). IL-17 is another inflammatory cytokine that has been implicated in the development of TA (142, 143). Both TNF and IL-17 activate similar cell signaling pathways. However, IL-17 by itself has little effect on inflammatory “activation” of ECs but it synergizes with TNF to up-regulate cell adhesion molecules and facilitate leukocyte transendothelial migration (144). Graft infiltrating Th17 cells have been associated with increased chronic allograft failure and lymphoid neogenesis in kidney transplantation, suggesting that Th17 responses that produce IL-17 could augment alloimmune responses locally within the graft (145). Although TA is a component of chronic kidney graft failure, other pathogenic mechanisms are also involved so future studies need to be performed to determine the potential effect of Th17 cells on lymphoid neogenesis as it relates specifically to the development of TA.

### Antibody-mediated alteration of graft vascular cell phenotypes

Due to their polymorphic nature and cell surface abundance, donor HLA molecules are a major class of antigens recognized by antibodies in graft recipients (146, 147). The presence of HLA reactive antibodies predicts the development of TA and studies in mouse models show that anti-MHC class I antibodies are sufficient to induce the development of this vascular condition (148–151). These antibodies may drive the development of TA by triggering phenotypic changes, such as increased proliferation and migration, in endothelial and smooth muscle cells (66, 152). These phenotypic changes could lead to structural alterations in arteries that remodel the vessel wall (153). In addition, anti-HLA antibodies amplify the immunogenic properties of the endothelium by up-regulating cell adhesion molecules and von Willebrand factor, which facilitates immune cell transmigration into the arterial wall. Anti-HLA antibodies also amplify T cell responses toward allograft arteries by increasing the antigen-presenting capabilities of the endothelium through a complement-dependent mechanism (Figure 1A) (62, 69, 154). NK cells have also been implicated in the development of TA through an antibody-mediated mechanism (151).

Some graft recipients also develop graft-reactive antibodies toward non-HLA molecules. This likely arises from an aversion of tolerance stemming from broad-scale inflammation that triggers activation of autoreactive lymphocytes and/or that creates neo-antigens through the proteolytic cleavage of self-proteins. Non-HLA antibodies that target vascular cells bind to vimentin, a novel fragment of perlecan termed LG3, and angiotensin II type 1 receptor (51–53). Increased levels of antibodies reactive to all mentioned antigens correlate with poor graft outcome in humans and these antibodies induce or accelerate TA and/or vascular dysfunction in animal models (52, 155–157).

### Vasoregulatory dysfunction in allograft arteries

**Figure 2:**
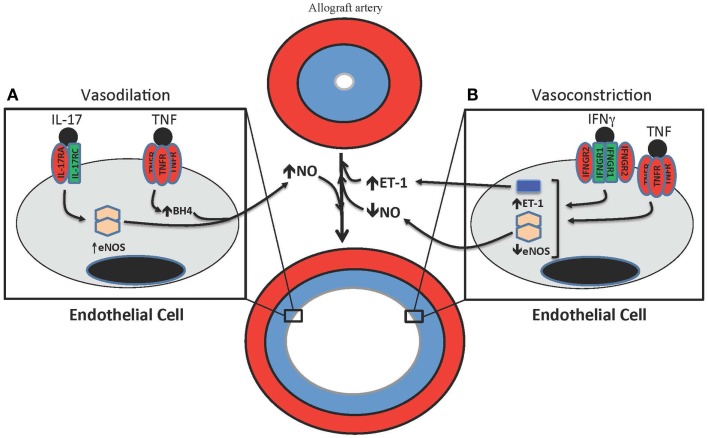
**T cell-mediated effects on vasodilation and vasoconstriction in allograft arteries**. **(A)** TNF and IL-17 are produced by T cells in allograft arteries. TNF acutely increases NO production from the endothelium by increasing eNOS activity through the up-regulation of tetrahydrobiopterin (BH_4_) synthesis. IL-17 increases NO production by increasing expression of eNOS. **(B)** IFNγ and TNF contribute to the vasoconstriction of allograft arteries by inhibiting the expression of eNOS, which reduces the levels of bioactive NO, as well as by increasing the production of the vasoconstrictive peptide ET-1.

Besides intimal thickening, another change in allograft arteries that ultimately drives ischemic graft failure is vasomotor dysfunction. The vascular endothelium is essential for regulating arterial vasomotor function, acting on vascular smooth muscle cells to control the dilation and constriction of blood vessels (158–160). The balance between vasodilatory and vasoconstrictive factors, as well as the inherent myogenic properties of the smooth muscle, determines blood flow through arteries. This balance is disturbed in allograft arteries (Figure 2) (161–163).

#### Immunological effects on vasodilation

Nitric oxide is an important endothelial-derived factor that induces arterial dilation (164). In blood vessels, this bioactive gas is produced by endothelial nitric oxide synthase (eNOS) expressed in the endothelium (165–168). Human Th1 effector CD4 T cells inhibit nitric oxide (NO) production from ECs by attenuating eNOS expression through the effects of both IFNγ and TNF (169, 170). This attenuation of eNOS expression by T cells compromises vasodilation, thereby providing an explanation for the early endothelial dysfunction observed after transplantation (171). Arterial dilation is also affected in allograft arteries by the production of NO by T cells that express inducible NOS (iNOS). NO production through this mechanism desensitizes smooth muscle cells to NO-mediated relaxation (170, 172, 173).

Th17 cells have been implicated in vascular changes that occur in TA (143), prompting us to investigate the effect of IL-17 on eNOS expression in ECs. IL-17 increased eNOS expression and NO production by human ECs through the coordinated activation of NF-κB, MEK1, and JNK signaling pathways. Further, eNOS expression was significantly correlated with increased levels of IL-17 in clinical specimens of TA and the abundance of this cytokine correlated with increased lumen size, but not intimal thickening, and increased eNOS expression (174). These data suggest that IL-17 could support outward arterial expansion in TA although a study examining the effects of IL-17 neutralization in a humanized model of allograft artery rejection did not observe any effects on total vessel area diameter (22). Also, IL-17 has been reported to reduce eNOS activity in mouse ECs and to contribute to hypertension in mouse models (175, 176). Additional studies are needed to determine the exact role of IL-17 in vascular changes and how this relates to TA.

Endothelial regulation of vasodilation is dependent on NOS co-factor availability. The “uncoupling” of eNOS that occurs when co-factors are limiting results in reduced NO bioactivity and a concomitant increase in reactive oxygen species (177). Both effects exacerbate pathological constriction of arteries in the presence of immunological responses. Interestingly, stimulation of human ECs with TNF, IL-1, or IFNγ augments eNOS activity by increasing tetrahydrobiopterin (BH_4_) levels through the induction of GTP cyclohydrolase I, the rate limiting enzyme in the BH_4_ synthesis pathway (178, 179). T cells also express GTP cyclohydrolase I and produce BH_4_ after activation through the T cell receptor (180, 181). It seems paradoxical that TNF and IFNγ both inhibit eNOS expression and increase its enzymatic activity, but it is possible that the induction of BH_4_ biosynthesis by these cytokines may support the recovery of endothelial-dependent vasodilation during inflammation (182).

#### Immunologic effects on vasoconstriction

The production of endothelium-derived constrictor factors (EDCFs) is also a key regulatory component of arterial vasomotor function. A discussion of EDCFs tends to be focused on endothelin (ET-1) since it is one of the most potent endogenous vasoconstrictors produced in humans and there are clear clinical indications for pathophysiological roles of ET-1, including in TA (183–186). TNF, IL-1, IFNγ, and IL-6 induce ET-1 production by ECs (187, 188).

Small arteries in humans and other mammals exist in a state of partial constriction. This allows a basal level of endothelial-independent arterial tone against vasodilators and vasoconstrictors that can act to influence arterial diameter and resultant blood flow. Allogeneic immune responses induce smooth muscle cell death in the media of allograft arteries and this likely reduces the functional properties of arteries (107). In a rat model of TA, myogenic constriction and dilation were both compromised at late time-points indicating that immune-mediated medial damage and dysfunction are prevalent (189). The loss of pressure-induced myogenic constriction in cardiac allografts could increase intravascular hydrostatic pressure and concomitant fluid leakage into the interstitial space, leading to loss of ventricular compliance and organ failure. Immunosuppression with cyclosporine reduces medial smooth muscle cell death and preserves myogenic activity (190).

## Cytoprotective and Immunoregulatory Features of the Graft Vasculature

The expression of cytoprotective and immunoregulatory proteins by tissue cells is essential for preventing pathological tissue damage and resultant immunopathology that can occur during immune responses toward pathogens. Similar processes may also be operational in a transplant setting and, as such, are pertinent to any discussion of alloimmune-mediated vascular injury. Profiling gene expression in non-rejecting or tolerized grafts has identified the increased expression of “cytoprotective” genes A20, hemeoxygenase-1 (HO-1), Bcl-xL, and Bcl-2 in non-rejecting grafts, suggesting a role for them in accommodating graft survival (191, 192).

A20 is a TNF-inducible zinc finger protein expressed by ECs and vascular smooth muscle cells (193). It inhibits EC death induced through both death receptor- and mitochondria-mediated mechanisms by preventing caspase activation and cytochrome *c* release from the mitochondria (194). As such, A20 may be able to prevent most forms of immune-mediated EC death that are operational in allogeneic responses. In addition to being cytoprotective in ECs, A20 is also able to reduce inflammatory responses by inhibiting NF-κB activation (195). This prevents the up-regulation of cell adhesion molecules and chemokines in ECs that facilitate leukocyte transendothelial migration into allografts. In vascular smooth muscle cells, A20 is anti-inflammatory through the inhibition of NF-κB activation but it also promotes cell death of intimal vascular smooth muscle cells, which is in contrast to its cytoprotective effects in ECs (196). All together, the overlapping but distinct functions of A20 in ECs and vascular smooth muscle cells may inhibit intimal thickening by preventing EC damage and ameliorating vascular smooth muscle cell accumulation in the intima. Indeed, A20 expression in vessel wall cells correlates with protection against TA and overexpression of A20 in donor artery segments in a mouse model of TA reduces the development of this vascular occlusive condition (197, 198).

HO-1 is an enzyme that catalyzes the conversion of heme to free iron, biliverdin, and carbon monoxide (199). It is expressed in several cells, including ECs, and has cytoprotective and immune-inhibitory effects. In transplantation, some studies have shown an association of HO-1 gene promoter polymorphisms with better kidney graft function and survival, although others have failed to observe this association (200–202). There is no apparent association between HO-1 gene promoter polymorphisms and TA in cardiac transplants (203). In preclinical studies, HO-1 in allografts has been shown to mediate graft survival (204). Importantly, the expression of HO-1 in vascular cells prevents the development of TA (205, 206). Experimental evidence further indicates that HO-1 prevents EC death, inhibits inflammatory responses, and attenuates adaptive immune responses (207). These effects are mediated through the actions of CO and biliverdin, which can prevent NF-κB and NFAT activation and may induce activation-induced cell death of T cells (208, 209).

In addition to the cytoprotective genes described above, graft vascular cells can also be induced to express immunoregulatory genes that are known to play a role in tolerance induction. These include IDO, programed cell death ligand-1 (PD-L1), and immunoglobulin-like transcript-3 and -4 (ILT3/4). IDO is an enzyme that degrades tryptophan, resulting in the release of kynurenines. The depletion of local levels of tryptophan can inhibit the proliferation of immune cells and kynurenines can actively inhibit immune cell activation (210). The induction of IDO by IFNγ in both ECs and vascular smooth muscle cells inhibits the activation of allogeneic T cells (211). IDO expression in ECs is also responsible for the development of cardiac allograft tolerance induced by the deoxyspergualine analog LF15-0195 in a rat model and its expression in vascular smooth muscle cells prevents alloimmune-mediated medial damage (32, 212). PD-L1 is also induced by IFNγ in ECs and vascular smooth muscle cells, and it inhibits the activation of effector T cell responses by binding to PD-1 on T cells (24, 213). The PD-L1/PD-1 system is an essential component of peripheral tolerance as indicated by the development or exacerbation of autoimmune-like manifestations in mice that lack PD-1 (214). In allografts, the expression of PD-L1 on arterial ECs reduces the development of TA (26, 215). Finally, Sucia-Foca and colleagues have identified the up-regulation of ILT3 and ILT4 on human ECs by CD8 T suppressor cells and IL-10, and established that these molecules inhibit allogeneic T cell activation (28, 216). The mechanism by which ILT3/4 on ECs is immunoregulatory remains to be fully elucidated but may involve the inhibition of T cell co-stimulatory signals (29).

## Therapeutic Opportunities in TA

Therapeutic prevention of TA requires a combination of strategies to specifically inhibit immune responses and to directly prevent hyperplastic responses of vessel wall cells. Non-specific immunosupression with cyclosporine is a mainstay in heart transplantation but the eventual failure of most grafts and the association of this drug with side effects necessitate the need for improved approaches. The “holy grail” of transplantation therapy is the induction of tolerance to specifically prevent immune activation toward transplanted grafts while maintaining protective immunity. Many preclinical studies demonstrate the induction of tolerance and prevention of TA using co-stimulatory blockade (217–219). In clinical studies, co-stimulatory blockade with CTLA4-Ig is effective for preventing kidney graft rejection although functional tolerance does not appear to develop (220). Similar studies have not been performed in heart transplant recipients to evaluate TA specifically. Clinical trials also suggest that it may be possible to induce tolerance toward kidney grafts by concurrently performing bone marrow transplantation that induces transient chimerism, although studies in non-human primates show that this approach may not lead to tolerance of heart grafts (221–223). A promising approach for the specific prevention of TA by immunomodulation has been established in a humanized mouse model of arterial rejection in which the delivery of *ex vivo*-expanded regulatory T cells, which suppress effector T cell responses, can prevent arterial remodeling reflective of TA (224, 225).

In addition to preventing immune activation, anti-proliferative drugs such as mTOR inhibitors inhibit smooth muscle proliferation and resultant intimal thickening in TA. These inhibitors first found use in the prevention of intimal hyperplasia after restenosis (226, 227). Studies in heart transplantation subsequently established that mTOR inhibition with everolimus reduced immune activation as well as intimal thickenining in TA through inhibiting smooth muscle cell proliferation in allograft arteries (228). The inclusion of everolimus in the immunosuppression regimen can reduce the dose of cyclosporine needed and, as a consequence, reduce cyclosporine-associated renal damage (229). Other studies have examined the *ex vivo* modification of allograft artery cells using viral transduction for the prevention of TA since it is likely that inhibition of vascular cell death by forced expression of protective genes, such as A20, can reduce the development of TA in preclinical models (198). Although such strategies seem ideal for therapies involving the *ex vivo* modification of graft cells prior to transplantation, such therapeutic approaches yet to be translated into a clinical setting.

## Conclusion

Blood vessels possess unique immunological features that define the outcome of immune responses. Cytotoxic damage of vessel wall cells and the alteration of vascular cell phenotypes by different components of the allogeneic immune response drives the remodeling of arteries in transplanted organs and this culminates in the development of TA. Further understanding these pathogenic mechanisms will be essential for future advancements that are able to specifically prevent immune activation toward allograft blood vessels and the hyperplastic responses of vessel wall cells. Also, similar immunopathological mechanisms that contribute to the development of TA are also involved in other immune-mediated arteriosclerotic conditions, such as giant cell arteritis and atherosclerosis, so insights obtained from studies on TA could also be informative for these diseases (76, 230).

## Author Contributions

Anna Von Rossum, Ismail Laher, and Jonathan C. Choy wrote the manuscript.

## Conflict of Interest Statement

The authors declare that the research was conducted in the absence of any commercial or financial relationships that could be construed as a potential conflict of interest.

## Abbreviations

AMR, antibody-mediated rejection; APC, antigen-presenting cell; EC, endothelial cell; EDCF, endothelial-derived constrictor factors; EDRF, endothelial-derived relaxation factor; eNOS, endothelial nitric oxide synthase; ET-1, endothelin-1; HLA, human leukocyte antigen; IDO, indoleamine 2,3-dioxygenase; IFNγ, intergeron-γ; ILT, immunoglobulin-like transcript; MHC, major histocompatibility complex; NO, nitric oxide; PD-1, programed cell death-1; PD-L1, programed cell death ligand-1; TA, transplant arteriosclerosis; TNF, tumor necrosis factor.
